# Discussion by Galveston County Medical Society

**Published:** 1893-01

**Authors:** 


DISCUSSION ON THE TREATMENT OF TYPHOID FEVER.

The President, Dr. Fly, referring to the history of typhoid fever, said that in 1878-79 the disease was almost unknown. In 1882 he had his first case. A boy, the son of the janitress in the

Hospital, had an attack of what some would call or describe as catarrhal fever (he did not belong to the class who described all cases of fever with intestinal symptoms as catarrhal). The boy had a temperature ranging from 102° to 104° F., with hemorrhage from the bowels, and died in three weeks from collapse. No post-mortem was obtained. The speaker believed this was a case of typhoid fever. His next cases occurred in 1884, when two patients came under his observation. The first case exhibited continued fever for four weeks, with marked tympanitis; then the fever disappeared, to be followed by a relapse lasting for four weeks longer. He believed it a case of relapsing typhoid. The second case was that of a conductor on the Santa Fe railroad. It was a typical one, with a distinct history of drinking from an infected well. In 1885, he had his next case, and since then he has had many other cases. Many cases that had been diagnosed as dengue, were accompanied by hemorrhage from the nose and bowels, and he believed they were really typhoid, as in typhoid fever there might be hemorrhage from any part of the alimentary tract. As to causes, he was convinced that they were due to the proximity of sunk cisterns to the cesspools, and quoted cases in point, both of typhoid and diphtheria. As to treatment, he considered nursing and diet of the first importance. He used predigested food a great deal, kept the bowels open with a daily enema, and treated pyrexia with cold baths. He did not believe in antipyretics, but treated high temperature with a cold bath (60° to 70° F.) to begin with, followed by ice-bags to the head and abdomen. He gave no solid food till two weeks after the disappearance of the fever. He believed the chief use of antipyretics was that they allowed the patient to die with a normal temperature. Later in the discussion, Dr. Fly said that he believed alcohol should be used freely when indicated. He insisted on frequent changing of the linen, and sponging with alcohol, to prevent bed sores. He objected to phenacetine. In private practice he gave the first cold bath himself, trusting the following one to a trained nurse. He commenced with a temperature of from 60° to 70° F. and applied iced water to the head and spine. Cold bathing was especially useful in averting convulsions in the hyperpyrexia of children. He did not believe that typhoid fever could be aborted by any treatment.

Dr. Randall felt that Dr. Fly had covered the grounds so thoroughly that little was left to be said. He considered that, setting aside preventive treatment, diet and hygiene were of the first importance and all drugs occupied a secondary place. When fever rose above 102.5° F., even before the diagnosis was made, he employed cold bathing, and repeated the bath every three hours, if necessary, commencing with a temperature at 80° F., and reducing it to 70° F., or lower, by addition of cold water or ice. He had the bodily temperature taken every two hours, and often made a morning bath a routine treatment. In his ward, at the John Sealy Hospital, he had had a portable bath tub introduced, and the Brand system thoroughly carried out. He found the patients were refreshed and rested much better after the bath, and the course of the fever was much modified. He considered such complications as pneumonia, bronchitis and pleurisy as contra-indications to the use of the bath. Hemorrhage, on the other hand, was a contra-indication, not because the cold contracted the capillaries and drove the blood to the internal organs, but because the exertion the bath entailed must be avoided; for the same reason the bath should not be used in threatened perforation. In the "typhoid state" he used turpentine in from five to ten drops every three or four hours. In persistent diarrhœa of convalescence, indicating an unhealed ulcer, he also found useful (as recommended by George B. Wood) the ingestion of the turpentine. He was aware that Dr. Osler had recently condemned the use of this drug, but was sure he had seen excellent results from its administration. He likewise used alcohol from the beginning of the disease as it was both a food and a heart stimulant. When there was failure of the circulation, he placed much faith in strychnine, carbonate of ammonium and ether. Digitalis should be used with care, as the heart muscle was often in a state of fatty degeneration dependent on myocarditis. He treated hemorrhage by absolute rest, opium, ergot, and the numerous astringents. Later, Dr. Randall spoke of the importance of efficient nursing as the only means of preventing bed-sores. He emphasized the importance of constant friction to the extremities and cold application to the head during the bath. He found no intestinal antiseptics of any use, except bismuth subnitrate and turpentine. He had used thymol, salol and naphthaline, without benefit. Salol he regarded as a dangerous remedy, as there was always a tendency to kidney mischief in typhoid fever, albuminuria being a frequent accompaniment of the disease. The drug, as was well known, was decomposed by the alkaline juices of the small intestine into phenol and salicylic acid, and these being eliminated by the kidney would increase the already existing trouble. He agreed with Dr. Cerna in considering musk in from 10 to 20 grain doses, by enema, as recommended by Dr. H. C. Wood, the best remedy in nervous exhaustion.

Dr. Cerna agreed with all that had been said about diet, hygiene, and the cold bath in pyrexia. In the "typhoid state" he believed that turpentine was of the greatest value. The drug was a stimulant, and exercised a local healing action on the ulcers. He believed in certain intestinal antiseptics, and recommended salol and the sulpho-carbolate of zinc for that purpose, in doses of from 5 to 10 grains of the salol and in from 2 to 5 grains of the zinc carbolote, alternately, every four hours. He insisted on the careful watching for a smoky color of the urine, when the salol should be stopped. He considered salol contra-indicated in Bright's disease, but if there were no intercurrent Bright's disease, he did not consider that any harm had been done, though the urine were rendered smoky, provided the drug were suspended at once. He recognized cases where cold bathing was useless to reduce temperature, every temporary reduction being followed in these cases by a rapid return to the previous thermic condition. These phenomena were due to exhaustion of the heat regulating centre, and he would like especially to draw attention to the use of musk as a nervous stimulant in such cases. By stimulating and sustaining the heat regulating center, it acted as a true antipyretic, and superceded baths in all cases where the centre was exhausted, that is, where the cold bath was followed by a rapid return to the former fever height. Of course, the price of the drug precluded its use in all but the wealthier classes.

Dr. Clopton noted in comparing the literature of the past with that of the present day, that while there had been great advances made in the diagnosis of typhoid fever, there has been little advance attained in the treatment of the disorder. He thought that as a guide to treatment, Stokes could be as much relied on as Reynolds or other recent authorities. The physician, to treat typhoid fever rightly, should keep three points in view. 1. He should realize that the fever could not be aborted by any treatment whatever, and that all attempts to make it abort were certain to do great injury. 2. He should remember that it was essentially an exhausting disease, producing no evanescent exhaustion but trying the sustaining powers of the patient to the utmost. He should, therefore, from the first, feed his patient up to his digesting capacity, but not beyond it. Milk, he regarded as the most important article of diet, and varied it only that he might aid his patient's appetite. Broths and hydrocarbons were also to be used. 3. He considered it of vital importance to begin alcohol in some form early. He preferred Scotch whisky. Brandy was difficult to get pure, and it was not so readily retained by the stomach. He began alcohol early and continued it late. In typhoid fever it was a positive food capable of being absorbed freely without the necessity of digestion. With regard to medicines, these must not be given with the idea of aborting the disease, as in this case they would do much harm. Complications occurring in typhoid fever, such as pneumonia, must not be treated apart from the original disorder—the typhoid fever must never be lost sight of when treating the complication. In pneumonia of typhoid fever, he found the carbonate of ammonium of the greatest service. He gave chloroform in small doses to quiet restlessness, and when it failed, found morphine of the utmost value. He had no faith in cold baths in private practice, as nurses were sure to mismanage them, and they involved great exertion in the patient. The only intestinal antiseptic that he had used was thymol, and in a very extensive experience of typhoid fever he had been convinced that its use had greatly reduced the percentage of cases of marked tympanitis, hemorrhage and perforation, as compared with their occurrence in his practice before he had commenced its employment. It caused no digestive disturbances and was an intestinal disinfectant of slow solubility. He used phenacetine when the temperature rose above 103° F. He treated hemorrhage with morphine, ergot, local cold and injections of cold water. Perforation, he treated with morphine, but had never had a case of undoubted perforation recover. He recognized great difficulty in diagnosing many cases from typho-malarial fever, so called, and recognized the value of the microscope in malarial cases.

Dr. Thompson had of course little to say on the purely medical treatment of typhoid fever. He wished, however, to call attention to one or two points. He could not believe that intestinal antiseptics could reach the ulcers of typhoid fever in such a condition as to produce any local effect on them whatever. It must be remembered that the drug had, in most cases, to travel through eighteen feet of intestine before it reached the ulcer, and must reach it in such a state of dilution that it was contrary to all surgical experience that it could exert any germicidal influence. They might perhaps favor healing by their general action in improving the physical condition of the patient. He deprecated the habitual use of alcohol in typhoid cases and thought it only added fuel to the fire; but considered it most valuable to tide over a crisis and in medicinal doses during convalescence. He would urge the greatest care in the administration of solid food after the disappearance of the fever as the slightest indiscretion would cause colic and diarrhœa and a rise of temperature. Convalescent patients were especially liable to painful constipation, and he could speak emphatically of the value of an enema of 1 to 2 drachms of glycerine immediately before going to stool in producing a copious evacuation of the bowels.

Dr. Sampson said that so fully had the subject been ventilated that little remained to be added. He considered that no more important advance in therapeutics had been made in recent years than the systematic use of cold baths in the treatment of typhoid fever. While the fever could not be aborted, he felt sure that it could be greatly modified and many of the complications averted by this measure. As to a doubtful diagnosis between pneumonia and typhoid fever precluding the cold bath, this objection was not valid. He had witnessed convincing proof in the practice of Dr. S. Baruch, of New York, of the efficacy of cold baths in the treatment of pneumonia. The speaker further believed that turpentine passed through the whole alimentary tract unchanged acting as an antiseptic to the canal and exhibiting a healing effect on the ulcers.

Dr. Raugh drew attention to the peculiar tendency to the development of bed-sores in typoid fever, and thought the physician should be especially careful to prevent their occurrence.

In closing the discussion, Dr. H. A. West said: "I have been very much interested in this discussion, and have very little to add to what has been expressed this evening or to my remarks at a previous meeting of the Society. There are, however, a few points not mentioned to which I would call attention. In the first place, I am very glad to hear Dr. Fly make the statement as to his change of opinion in regard to those cases of fever occurring about 1885, and called by most of the physicians here typho-dengue. It will be remembered by Dr. Fly and others present here this evening, that when the nature of the fever prevalent in Galveston at that time was discussed at a meeting of the old Society, that I stood almost alone in advocating the view that typhoid fever was then epidemic in our city; contending for this view upon the grounds of the long duration of the disorder, the serious complications, such as intestinal hemorrhages, parotitis, etc., the characteristic course of the fever, and the very considerable mortality. It is gratifying to me to have my views upon these important questions not only endorsed by Dr. Fly, but by nearly all the physicians in Galveston. Now, let me present a few points in the treatment of typhoid fever, which have not been mentioned. 1. Let me insist upon the important attention to cleanliness and an antisepsis of the mouth and fauces. This is a matter which is too often neglected. The mouth should be systemetically and frequently cleansed by a solution of boric acid, carbolic acid, or the like, in order to get rid of decaying particles of food and septic germs of various kinds. We can accomplish much in preventing serious pulmonary complications, as bronchitis and pneumonia, by the employment of such a measure. While I use few drugs in the treatment of typhoid fever, there are some which may be of decided benefit, especially those which act by aiding digestion. If there is any disease where artificial assistance to digestion is demanded, it is the one under consideration, as during its course the digestive power of patients is reduced to that of an infant; hence the necessity of giving not only the most easily assimilated and nourishing foods, but also pepsin and hydrochloric acid to aid the weakened stomach in performing its functions. Pepsin not only acts as a digestant but as an antiseptic, and is of undoubted value. A word as to intestinal antiseptics. Theoretically they are all right, practically they often fail, and for various reasons: First, they may be absorbed and never reach the intestines, except in a state of extreme dilution in the blood; and secondly, because many of them cause nausea, vomiting and disgust for food, and any medicine which produces such effects should be interdicted. There is one antiseptic, however, which does not offer these objections, and which, in my opinion, really does good, namely, subnitrate of bismuth. The medicament should be given in large doses. A word as to antipyretics. Frequent reference has been made this evening to the effect of baths in the treatment of the fever. It should be remembered that cold bathing produces other and more important results than a mere reduction of the bodily heat. The cold bath is a good stimulant to the nervous centres, strengthening especially the respiration and circulation. It produces a soothing influence, promotes sleep, improves digestion and the appetite, stimulates the action of the skin, and, in fact, through its action upon the central nervous system, it is beneficial to the entire economy. When the physician is thoroughly convinced as to the truth of these statements (and one has only to try this treatment to be convinced), he will have the courage of his convictions and thus overcome all such difficulties as objections of friends, want of facilities, etc. As to the medicinal antipyretics, I do not believe in them; they are not only theoretically harmful, but practically so, as I have found by experience. I remember one case in which, owing to the persistent daily rise of the bodily temperature, I was tempted to employ (during the period of approaching convalescence) 7 grains of phenacetine. This single dose produced a condition of alarming collapse. I believe that all or any of the coal-tar antipyretics, if not absolutely contra-indicated in typhoid fever, should at least be used with great circumspection. I never use them at all, except perhaps at the very onset of the fever."

